# Plant Biostimulants from Cyanobacteria: An Emerging Strategy to Improve Yields and Sustainability in Agriculture

**DOI:** 10.3390/plants10040643

**Published:** 2021-03-29

**Authors:** Gaia Santini, Natascia Biondi, Liliana Rodolfi, Mario R. Tredici

**Affiliations:** Department of Agriculture, Food, Environment and Forestry (DAGRI), University of Florence, Piazzale delle Cascine, 18, 50144 Florence, Italy; gaia.santini@unifi.it (G.S.); natascia.biondi@unifi.it (N.B.); mario.tredici@unifi.it (M.R.T.)

**Keywords:** biostimulants, cyanobacteria, sustainable agriculture

## Abstract

Cyanobacteria can be considered a promising source for the development of new biostimulants as they are known to produce a variety of biologically active molecules that can positively affect plant growth, nutrient use efficiency, qualitative traits of the final product, and increase plant tolerance to abiotic stresses. Moreover, the cultivation of cyanobacteria in controlled and confined systems, along with their metabolic plasticity, provides the possibility to improve and standardize composition and effects on plants of derived biostimulant extracts or hydrolysates, which is one of the most critical aspects in the production of commercial biostimulants. Faced with these opportunities, research on biostimulant properties of cyanobacteria has undergone a significant growth in recent years. However, research in this field is still scarce, especially as regards the number of investigated cyanobacterial species. Future research should focus on reducing the costs of cyanobacterial biomass production and plant treatment and on identifying the molecules that mediate the biostimulant effects in order to optimize their content and stability in the final product. Furthermore, the extension of agronomic trials to a wider number of plant species, different application doses, and environmental conditions would allow the development of tailored microbial biostimulants, thus facilitating the diffusion of these products among farmers.

## 1. Introduction to Plant Biostimulants

As a result of the growing population, by 2050 the world will need 60% more food than is available today and about 80% of this increase will need to come from land that is already under cultivation [1]. This goal, together with climate change and the decline in the availability of natural resources, poses a serious challenge to agriculture. To meet the present food demand, agriculture makes heavy use of agrochemicals (synthetic fertilizers and pesticides), intensive tillage, and over-irrigation, which leads to pollution, high emission of greenhouse gases, and loss of essential ecosystem services [2,3]. On the other hand, the use of organic agriculture often results in yield losses of 20% and more compared to conventional cultivation [4] due to higher biotic pressure as well as nutrient limitation [5]. Therefore, biotechnologies that allow an efficient management of resources, in particular of water, nutrients, and soil, ensuring at the same time high yields and high-quality products, will be essential in the coming years for a sustainable intensification of agriculture [2,6].

In this context, plant biostimulants have received increasing attention from the scientific community and agrochemical industries over the past two and a half decades [7,8,9,10]. Biostimulants are meant to complement plant protection products and traditional fertilizers, as they are defined a “fertilizing product which function is to stimulate plant nutrition processes independently of the product’s nutrient content with the sole aim of improving one or more of the following characteristics of the plant or the plant rhizosphere: (I) nutrient use efficiency, (II) tolerance to abiotic stress, (III) quality traits, or (IV) availability of confined nutrients in the soil or rhizosphere” [11]. These characteristics of biostimulants derive from the action of bioactive compounds which are effective on plants at significantly lower concentrations compared to macronutrients [12].

The biostimulant market is one of the fastest-growing agriculture-related sectors, exhibiting a CAGR (compound annual growth rate) of 10.65% in the period 2019–2027, while the global inorganic fertilizer market, which is currently 60 times wider, is growing at a rate of 1.3–1.8% annually [13].

The biostimulant industry is investing significantly (between 3% and 10% of annual revenues) in research and development to meet the needs of this rapidly expanding market [8], not to mention public research investments. As a consequence, our knowledge of biostimulants and their beneficial effects has been improving at a considerable rate [14] and the number of scientific papers in this field has increased by about 30 times in the last three decades [15]. Currently, among the most relevant research topics, there is the standardization of biostimulant production processes in order to guarantee standard chemical and biological characteristics of the final product and reproducible effects on treated plants [14]. Since standardization starts with the selection of feedstock, this issue represents a critical aspect in the production of many biostimulants, where the raw materials are collected from natural environments (e.g., seaweeds) or derive from agro-industrial by-products [16].

The biostimulant effects of seaweed extracts are confirmed by numerous studies and commercial trials [16,17]. However, it is also widely known that the composition and content of active substances in seaweeds can be affected by many factors including tissue age, environmental conditions, nutrient availability, and time of harvesting [7,18]. In particular, polysaccharides, which are one of the major components of seaweed biomasses, have been reported to accumulate in brown seaweeds during summer as a response to increased temperature and irradiance, while consumption of polysaccharides may take place during winter [19,20,21]. Qualitative and quantitative changes related to season and growth stage were reported for endogenous cytokinins in *Ecklonia maxima* and *Sargassum heterophyllum* [22,23]. This compositional variability is reflected in the intensity and type of responses elicited on plants [24]. In addition, if seaweeds are collected from polluted waters, they can contain heavy metals (Al, As, Pb, Cd) in quantities exceeding those allowed by the European Union regulation 2019/1009 [25,26,27]. A high variability in the levels of beneficial phytochemicals (mainly phytohormones and phenolic acids), as well as the presence of heavy metals or biological contaminants, was also detected in commercial biostimulants derived from agro-industrial by-products, as the wastes used to generate these biostimulants are complex mixtures of substances that exhibit batch differences which can, in turn, affect their performance on plants [28,29].

Studies on the application of biostimulant products on different plants have highlighted that the effectiveness can vary in relation to the plant species and the cultivation conditions [9,30,31]. Among the possible causes, there is a lack of standardization but also the metabolic diversity of treated plants, as the sensitivity thresholds for one or more of the bioactive molecules in the product can vary among plant species or even among different cultivars of the same species [14]. In some cases, biostimulants obtained from animal residues through chemical hydrolysis of protein have even produced phytotoxic effects, such as growth suppression, due to the high content of free amino acids and D-amino acids [32,33]. To strengthen the credibility of biostimulant products among farmers, it is necessary to deepen our knowledge of the mechanisms underlying the observed effects and develop more reliable and tailored products for different plants.

## 2. Agricultural Use of Cyanobacteria: From Biofertilizers to Biostimulants

**Figure 1:**
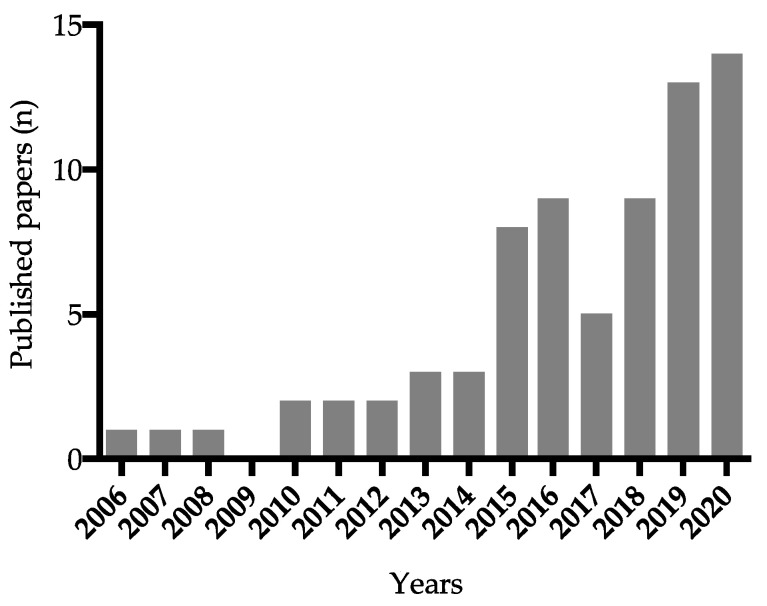
Research articles per year published on biostimulants from cyanobacteria since 2006. The bibliometric analysis was carried out using the Google Scholar database, searching for “cyanobacteria + biostimulants”, “microalgae + biostimulants”, and “cyanobacteria + plant growth promotion” and excluding articles where the action was predominantly as biofertilizer.

Cyanobacteria are ubiquitous in nature and their presence has long been reported in different soils (agriculture soils, rice fields, mines, desert lands, marshy soils) where they are responsible for bringing positive effects in different ecological situations [34,35]. Since the 1950s, application to soil of dry biomass (bio-inoculation) of different cyanobacteria, initially called “algalization”, has been shown to improve growth, health, and yields of various crops [36,37]. These beneficial effects were traditionally attributed to the supply of essential nutrients to plants and to the improvement of soil texture, structure, and water retention capacity [38,39,40]. Cyanobacteria can annually contribute about 20–30 kg N ha^−1^ thanks to nitrogen fixation [41] and can improve the bioavailability of phosphorus by solubilizing and mobilizing insoluble organic phosphates [42]. However, several studies suggest that increasing nutrient availability is not the only mechanism that contributes to promote plant growth in inoculated soils [36,39]. In fact, it has been confirmed that, besides natural fertilizing and balancing mineral nutrition, biologically active molecules secreted by cyanobacteria, including osmolytes, phenolics, proteins, vitamins, carbohydrates, amino acids, polysaccharides, and phytohormones, may work in synergy to promote plant growth [43,44]. The abilities of cyanobacteria to produce bioactive molecules, effective at low doses on plants, together with the possibility to obtain biomass with more constant chemical and functional characteristics, thanks to highly controlled cultivation conditions, has led the scientific community to focus on cyanobacteria and microalgae as a promising bioresource for the production of a new class of high-quality biostimulants [26,45,46]. Published papers on the in vivo biostimulant activity of cyanobacteria have increased since 2008, with a boost in 2015, and the general trend is towards a further increase (Figure 1).

**Figure 2:**
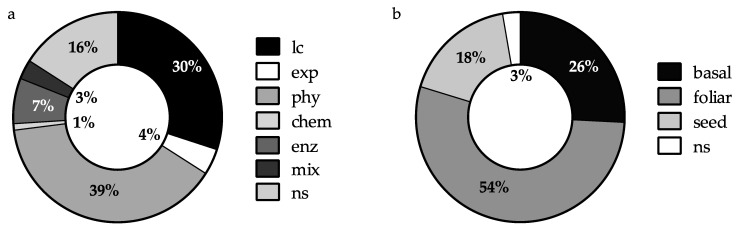
Cyanobacteria processing methods (**a**) and methods of application to plants (**b**) in the papers found through bibliometric analysis. The graphs represent the distribution (%) of publications in the period under review (2006–2020). lc, living cells; exp, released extracellular products; phy, biomass physical extraction; chem, biomass chemical extraction; enz, biomass enzymatic extraction; mix, mixed extraction (physical and chemical); basal, basal application; foliar, foliar spraying; seed, seed coating; ns, not specified.

In these studies, application and processing methods of cyanobacterial biomass are quite heterogeneous and include direct inoculation with living cells and different treatments, such as mechanical/physical extraction (e.g., autoclaving, drying and grinding, lyophilization, heating with water, sonication, supercritical CO_2_), chemical extraction (alkalis or acids), and enzymatic extraction (e.g., with proteases) (Figure 2a). However, in 39% of the experimental studies, cyanobacteria are administered to plants in the form of aqueous extracts obtained from dried biomass extracted with water after sonication or mechanical disruption of the cells (grinding with pestle and blender) (Figure 2a). Living cells are directly applied in 30% of the studies, while in 16% of the cases the production methods are not disclosed, being confidential information (Figure 2a). In very few studies (4%), extracellular products contained in the culture medium are applied (Figure 2a). Compared to the use of the whole biomass, in general, cell extraction improves plants response to treatment as it allows a higher recovery of the active compounds enclosed in the cell or linked to the cell wall [47,48]. It is widely accepted that the extraction method greatly influences composition and bioactivity of the extracts [49]. Mogor et al. [50] report that *Arthrospira platensis* hydrolysis with a protease resulted in a 34% increase in spermine and a 41% reduction in putrescine content in the hydrolysate compared to the non-hydrolyzed biomass. The effects of non-hydrolyzed biomass and hydrolysates at low reaction times (2 h) were similar or slightly lower than those of the control in in vivo trials on lettuce seedlings, while longer reaction times of hydrolysis (4 and 6 h) had a significant effect in promoting plant growth in terms of leaf area and roots and leaves fresh weights [50]. In the same study, enzymatic hydrolysates of *A. platensis* with high reaction times (4 and 6 h) showed lower auxin and higher cytokinin effects in cucumber cotyledon bioassays compared to *A. platensis* non-hydrolyzed biomass.

To deliver cyanobacterial biostimulants to crops, various application methods have been adopted (Figure 2b). The main mode of application is by foliar spraying (54%), followed by basal application (26%) (in soil or inert hydroponic substrates) and seed coating (18%). Inoculation with living cells is mainly applied basally or on the seed, while the extracellular products are applied either foliarly or added to the hydroponic medium. Since cell extracts applied to soil may not meet crop requirements due to physical, microbiological, and chemical characteristics of soil and rhizosphere, they are usually applied to plants by foliar spraying or seed coating [45]. Foliar spraying of organic substances is one of the most effective agricultural practices, as it limits dispersion of nutrients into the environment [51,52] and allows to use lower doses of product [53], thus improving the economic sustainability of treatments. Mahmoud et al. [54] showed that foliar application of an *Anabaena sphaerica* aqueous extract on spinach leaves was more effective in increasing plant dry weight, nutrient uptake (P and K), and protein content than soil application at equal concentrations (10 g L^−1^). Moreover, plant responses to nutrients and bioactive molecules present in the extracts are normally more rapid with foliar applications than with application as a soil amendment [17,55].

The results of the bibliometric analysis reported above indicate that research in this field is still very limited, especially as regards the number of investigated cyanobacterial species. The total number of scientific papers published from 2006 to 2020 is 73 (Figure 1), 49% of which concern *A. platensis*. Considering that the estimated number of existing cyanobacterial species ranges from 6280 to 8000, about 40% of which have been described so far [56,57], we can state that the genetic variability of these microorganisms is far from being explored. Cyanobacteria, therefore, represent one of the most promising sources of new products [36,39], especially for agricultural applications where their use is still scarce.

## 3. Cyanobacterial Biostimulant Characteristics Based on the Effects on Plants

In vivo studies on cyanobacterial biostimulants have highlighted various positive effects on plant growth and physiology, here divided according to the main effects produced (Table 1) and considering the characteristics that define plant biostimulants [11].

### 3.1. Nutrient Use Efficiency and Availability of Nutrients in Soil

Fertilizer use in modern agriculture is highly inefficient and most of the applied fertilizers are lost causing environmental pollution such as eutrophication of inland and coastal waters [93,94] or become unavailable to the crop through chemical, physical, or biological transformation [95]. Furthermore, the industrial production of chemical fertilizers is an energy-intensive process that significantly contributes to global CO_2_ emissions [96]. A way to reduce fertilizer use without affecting plant nutrition is to enhance crop uptake through the use of biostimulants [97]. Biostimulants can stimulate the absorption of nutrients by indirectly acting on soil structure and on availability of soil nutrients or by directly affecting plant physiology [7,97]. It is well known that cyanobacteria can produce extracellular polysaccharides which bond to metallic ions in the soil and produce a gel that helps to hold water and maintain soil aggregate stability [34]. Moreover, soil inoculation with cyanobacteria can lead to a significant increase of alkaline phosphatase activity next to the roots with beneficial effects on phosphorus mobilization [98]. Furthermore, these microorganisms are known to produce siderophores and increase iron concentration in the root zone, facilitating the uptake and translocation of iron in the plant [71,99]. Thanks to the crosstalk between iron and zinc uptake mechanisms in the plant, siderophores could in turn influence the mobility and translocation of zinc from plant roots [53].

The enhancement in nutrient uptake has been observed when cyanobacteria are applied to soil and by foliar spraying in the form of extracts. In the study of Anitha et al. [53], *A. platensis* was evaluated as a crop biofortification agent in *Amaranthus gangeticus*, *Phaseolus aureus,* and *Solanum lycopersicum*. Although all treatments stimulated the accumulation of zinc, foliar application was effective at lower concentrations (10 and 15 g L^−1^) compared to seed coating (100–300 g L^−1^) and to soil application. Tarraf et al. [87] showed that fenugreek plants sprayed with an extract of *A. platensis* at two different concentrations (2.5 and 5.0 g L^−1^) presented higher N, P, and K contents compared to the untreated plants. Treatment with the higher concentration (5 g L^−1^) produced a significant increase in plant growth. *A. platensis* applied foliarly in the form of a protein hydrolysate at 10 g L^−1^ enhanced foliar concentration of N, P, K, Ca, and Mg in petunia [84]. This resulted in higher root dry weight (+35%), flowers number (+66%), and weight (+20%) and earliness of flowering in treated plants compared to the controls [84].

The concentration of an algal biostimulant appears to be a critical factor for its effectiveness [100]. Seaweed extracts are known to be active at very low concentrations (diluted 1:1000 or more), especially when compared to biofertilizers [101]. This also happens with cyanobacterial extracts, where the application of high doses can nullify the biostimulant effect. Godlewska et al. [83] found that the enhancement in radish fresh weight after foliar spraying with *A. platensis* growth medium positively correlated with the applied doses up to a maximum concentration of 15%, beyond which a decreasing biostimulant activity was observed. Micronutrients content in radish followed the same trend, with the higher content of B, Cu, Fe, Mn, Ni, and Zn obtained following the application of 20% *A. platensis* growth medium, while higher concentrations generally caused a decrease in the microelements content [83].

In the case of foliar application of *A. platensis* biomass on red beets, the effective concentration has been reported to change with cultivar [86]. The Early Wonder and the Scarlet F1 cultivars presented similar improvements in fresh and dry weight (ca +65%) at the highest tested concentration (3 g L^−1^), while a lower concentration (1.5 g L^−1^) was effective only on the Early Wonder cultivar (ca +50%), indicating different effects of the same dose as a function of plant genotype. In the latter mentioned study, the authors correlated the bioactivity observed on the plant with a high content of free amino acids in the applied biomass. *A. platensis* is widely recognized as a rich protein source, with a protein content up to about 70% in the dry biomass [102]. The enzymatic hydrolysates obtained from *A. platensis* have been shown to contain valuable biocompounds including L-amino acids, amounting to about 60% of total protein content [103,104]. The supply of L-amino acids is considered beneficial in plant nutrition as they are directly used by plants for protein synthesis [86] and their exogenous application has been reported to increase nutrient uptake [105]. In contrast, D-amino acids can produce phytotoxic effects when supplied at similar concentrations [106]. D-amino acids are abundant in protein hydrolysates obtained by chemical hydrolysis, while enzymatic hydrolysis leads to a low racemization rate [6].

The use of cyanobacteria, applied in different forms on both roots and leaves, has been shown to positively affect root development and root/shoot ratio in various plant species, such as lettuce [50], radish [83], rice [43], tomato [47,80], peppermint [61], strawberry [107], maize [108], red beet [86], cucumber [99], and petunia [84]. A larger root system increases the root surface area and directly improves nutrients and water uptake from soil, thereby enhancing plant growth and vigor [59]. These effects are attributed to phytohormones, especially auxins, that are able to affect root development by both improving lateral root formation and increasing the total volume of the root system [12,109]. This is confirmed by the fact that cyanobacteria can be rich in auxin and auxin-like compounds, whose concentration greatly varies from about 2 μg g^−1^ (*Anabaena vaginicola*) to about 40 μg g^−1^ (*Leptolyngbya* sp.) according to the species and the analytical technique adopted [44,110]. It has also been reported that high levels of abscisic acid suppress ethylene synthesis, which in turn reduces auxin transport and biosynthesis in the root tip, thereby promoting root elongation [111]. Abscisic acid was found to be 0.59 and 0.68 μg g^−1^ in *Cylindrospermum muscicola* and *Anabaena oryzae*, respectively [62]. Moreover, cytokinins enrichment in plant roots can cause an enhancement in the expression of genes encoding for root nitrate and sulfur transporters [112,113], thereby increasing plant nutrient uptake. In this regard, Mutale-Joan et al. [59] measured NPK concentrations in roots to evaluate the effects on nutrient uptake of 18 crude extracts obtained from microalgae and cyanobacteria, applied three times as soil drench on tomato seedlings. The highest root dry weight (+35%) and root length (+113%) increase over controls were obtained with an *Aphanothece* sp. extract. This treatment also displayed the maximum N, P, and K uptake, which increased by 101, 65, and 78% compared to untreated plants. Principal component analysis confirmed that improved P and K levels in roots were closely associated with enhanced root length, while root N concentration was more closely associated to shoot dry weight and chlorophyll content in leaves, indicating a correlation between nitrogen uptake, photosynthetic activity, and shoot biomass accumulation in treated plants [59].

Since part of the nitrogen taken up by roots is invested in photosynthetic machinery, an improved nutrient uptake can strongly affect plant photosynthetic rate and consequently plant growth [114]. Haroun and Hussein [62] reported that seed priming with culture filtrates of *C. muscicola* and *A. oryzae* led to an increase in chlorophylls in lupin leaves, consequently increasing the photosynthetic activity and carbohydrate content in the shoot. Many other studies reported increased chlorophyll content in plants treated with cyanobacterial extracts; such effect can be attributed to a better nutrient use efficiency by plants or to a protective effect of the biostimulant, that reduces chlorophyll degradation and delays plant senescence [47,59,80,90]. Exogenous application of amino acids is known to stimulate nitrogen metabolism efficiency and synthesis of chlorophylls in treated plants [86]. Phytohormones such as cytokinins, betaines, and gibberellins in cyanobacterial extracts may play a role in reducing chlorophyll degradation mainly through inhibition of chlorophyllase activity [115,116]. Among substances with hormone-like activity, exogenously applied polyamines can be covalently conjugated to chlorophyll-bound proteins by plastidial transglutaminases, thus improving chlorophyll stability during leaf senescence [117]. These findings suggest a role for cyanobacterial applications in mitigating the negative effects of abiotic stresses on crops.

### 3.2. Tolerance to Abiotic Stresses

The reduction in yield and crop quality due to abiotic stresses, such as drought, salinity, and extreme temperatures, drastically limits agricultural production in many parts of the globe [118]. These hindrances are expected to worsen in the coming years due to climate change which is causing an unprecedented increase in extreme weather events and patterns [17]. Despite the urgent need, strategies to mitigate abiotic stresses are still scarce [17]. Cyanobacteria can promote plant growth and development in stressful conditions by producing and releasing a diverse array of biologically active molecules, which can induce systemic responses in plants to fight against stresses [44]. These signaling compounds, known as elicitors, can affect plant gene expression and stimulate the accumulation of a wide range of phytochemicals (e.g., glucosinolates, alkaloids, polyphenols, flavonoids, flavonoid glycosides, saponins, terpenes, phytoalexins), which provide protection to the plant towards biotic and abiotic stresses [44,119].

Rice plants in pots inoculated with various cyanobacterial strains showed consistent accumulation in leaves of phenolic acids (gallic, gentisic, caffeic, chlorogenic, and ferulic acids), flavonoids (rutin and quercetin), phytohormones (indole acetic acid and indole butyric acid), proteins, and chlorophylls [43]. The increase in the levels of phytochemicals is an indicator of enhanced gene expression in plants induced by elicitor molecules and is positively correlated with an increase in germination rate, shoot length, root length, and biomass in inoculated plants [44]. Effects on plant metabolism were also observed with the application of cell extracts. Mutale-joan et al. [59] found that treatment of tomato seedlings with cyanobacterial extracts significantly influenced the profile of several lipophilic metabolites related to plant abiotic stress tolerance. The highest phytol (+2044% and +1973%) and phytyl fatty acids (+1088% and +1008%) content enhancement compared to untreated controls was recorded after treatments with *Aphanothece* sp. and *Arthrospira maxima*. Phytol (mostly derived from chlorophyll degradation) [120] is an essential precursor in the biosynthesis of tocopherols which are well-known lipid antioxidants that contribute to the protection of photosystem II against photodamage [121,122]. In chloroplasts of *Arabidopsis thaliana*, a large proportion of phytol and fatty acids is converted into fatty acid phytyl esters, which are involved in maintaining the integrity of the photosynthetic membrane during abiotic stress and senescence [123]. Plants treated with cyanobacterial extracts resulted also in the accumulation of linolenic acid, a key precursor in the biosynthetic pathway leading to jasmonates, in a considerable amount (+673% and +561% with *Aphanothece* sp. and *A. maxima*, respectively) compared to the untreated controls [59].

Many abiotic factors (drought, salinity, extreme temperatures) are manifested in plants as osmotic stresses, leading to accumulation of reactive oxygen species (ROS) that damage DNA, lipids, carbohydrates, and proteins and also cause aberrant cell signaling [100]. Soil inoculation or foliar application of cyanobacterial-based biostimulants have been shown to strengthen the antioxidant activity of treated plants, thus mitigating the effects of stress-induced free radicals by direct scavenging and preventing ROS formation [43,67,89,108]. Singh et al. [43] reported that soil inoculation with *Oscillatoria acuta* and *Plectonema boryanum* induced systemic tolerance against stress by enhancing enzymatic activity of peroxidase and phenylalanine ammonia lyase in rice leaves, while total phenolic content reached maximum values after inoculation with *A. oryzae*.

Although evidence is accumulating on the potential of cyanobacteria in eliciting defense responses in plants, characterization of the elicitor molecules and of their mechanisms of action is still poor, which hampers their possible use as plant “pre-stress conditioners” [7,44]. However, recently remarkable progress has been made in our understanding of elicitors from cyanobacteria.

#### 3.2.1. Phytohormones

The inhibitory effect of abiotic stresses on plant growth is exhibited at several levels and involves an array of cellular processes that are regulated by hormones for which homeostasis may be altered during stress [124,125]. Therefore, the exogenous application of different growth regulators contained in or released by cyanobacteria can increase the resistance of plants to environmental factors by enhancing endogenous hormone levels [58,126]. Recent findings suggest that the ability of cyanobacteria to promote plant growth is not only linked to their hormone content, but also to their capacity to stimulate endogenous hormone synthesis in treated plants [126]. It has been demonstrated that the endogenous auxin content of wheat inoculated with cyanobacteria is significantly correlated with exogenous auxin production by cyanobacterial strains [127]. Moreover, it was observed that cyanobacteria produce more endogenous and exogenous auxins in the presence of a plant, suggesting that plants might release signals responsible for higher auxin production [127].

Rodriguez et al. [58] hypothesized a correlation between the production of gibberellin-like substances and the ability of *Scytonema hofmanii* extracellular products to partially counteract many of the NaCl-induced effects on growth of rice seedlings, in particular on reduction of shoot length, root dry weight, and total free porphyrin content. Bayona-Morcillo et al. [91] applied an enzymatic protein hydrolysate of *A. platensis*, rich in cytokinins, on leaves of petunia exposed to 2.0, 2.5, and 3.0 dS m^−1^ EC (electrical conductivity). Increasing salinity progessively increased leaf concentration of Na^+^ and Cl^−^ and decreased K:Na ratio, causing deleterious effects on plant growth. However, following the application of *A. platensis* hydrolysate, an increase in the number of leaves, shoots, and flowers and in K:Na ratio was found in plants subjected to the highest salinity compared to the untreated controls. Aqueous extracts and hydrolysates of *A. platensis* have been shown to contain, in relatively high amounts, phytohormones, such as abscisic acid, cytokinins, and jasmonic acid, involved in plant response to abiotic stresses [91,128]. Production of abscisic acid in response to salt stress was also observed in the culture medium of *Nostoc muscorum* and *Synechococcus leopoliensis* two hours after NaCl application [129]. The presence of salicylic acid, an important signaling molecule responsible for the activation of defense responses in plants, was detected in the cellular extracts of 28 cyanobacterial strains. Quantitative analysis through ELISA immunodiagnostic tests revealed that *Calothrix* SAB-B797 and *Nostoc* SAB-M612 were the richest strains in salicylic acid with contents of about 85 μg g^−1^ [99].

Even if it is well documented that cellular extracts and growth media of several cyanobacteria contain phytohormones [44], other biologically active substances such as vitamins, amino acids, and polysaccharides may act as precursors or activators of endogenous plant hormones, thereby exhibiting hormone-like activities on plants [7,130].

#### 3.2.2. Amino Acids and Polyamines

Besides their role in plant nutrition, L-amino acids can function as signaling molecules to mitigate injuries caused by abiotic stresses [131]. Recent reports indicate that melatonin, derived from L-tryptophan via the shikimate pathway, can prime seeds to tolerate adverse environmental conditions at the germination stage [132]. The application of *A. platensis* lyophilized biomass, which is known to be rich in L-amino acids, has been reported to stimulate red beet carbon metabolism, chlorophyll synthesis, and sugar content [86], also providing beneficial effects during stress. The improved chlorophyll synthesis may be linked to the synthesis of aminolevulinic acid that can derive from the carbon skeleton of exogenously applied L-glutamic acid [133].

*A. platensis* enzymatic protein hydrolysates contain polyamines [50]. Other cyanobacteria (*Synechocystis* spp., *Anabaena* spp.) have been shown to accumulate polyamines under stressful conditions [134]. Polyamines are a group of low molecular weight aliphatic amines whose synthesis occurs from the decarboxylation of L-amino acids such as L-arginine and L-ornithine [50]. In bacteria and plants, polyamine synthesis is related to the ability of these organisms to tolerate different types of environmental stresses [135,136] as they are involved in a number of osmoprotectant functions such as nucleic acid protection, regulation of gene expression and protein synthesis, modulation of signal transduction, cell membrane stabilization, and prevention of chlorophyll loss during leaf senescence [137,138]. In particular, spermine and spermidine have been indicated as the bioactive compounds responsible for growth enhancement in lettuce plants treated with enzymatic hydrolysates of *A. platensis* [50]. Cyanobacterial treatments not only increased lettuce dry weight by about 40% compared to the untreated controls but also increased spermine content in plant leaves by 64%, suggesting growth-promoting effects and plant signaling ability of these bioactive molecules [50].

#### 3.2.3. Polysaccharides

Polysaccharides are among the most versatile molecules extracted from algae, providing a broad range of applications in medicine (antibacterial, antioxidant, anti-inflammatory, antitumoral, and antiviral properties), food, and pharmaceutics (cosmeceuticals, nutraceuticals) [139,140,141]. Algal polysaccharides have been also shown to display a number of biological activities on higher plants, including the ability to elicit defense responses [142,143], and are one of the major components of commercial seaweed extracts accounting for up to 30–40% of extract dry weight [144]. Although seaweed polysaccharides have been largely exploited as plant defense inducers, to date little attention has been addressed to cyanobacterial and microalgal polysaccharides as plant biostimulants [145]. Cyanobacterial polysaccharides can be incorporated in the cell-wall, excreted as definite structures (sheaths, capsules, or stalks), or released as mucilage [146]. In particular, exopolysaccharides have been reported to play a significant role in soil aggregation due to their gluing properties [39] and in binding heavy metals [147] and sodium ions [148], thus improving plant development in saline or polluted soils. Seifikalhor et al. [92] applied *A. platensis* as a maize seed coating, observing a reduction in Cd uptake thanks to polysaccharides binding of Cd ions, thus mitigating the toxic effects on plants. The reduction in roots Cd content of seed-coated plants was more than 90% after 12 days from sowing.

In the last few years, evidence on phytostimulant properties and plant signaling abilities of cyanobacterial polysaccharides applied on plants has been accumulating [80,81,149]. *A. platensis* crude polysaccharides were extracted by heating biomass at 90 °C for four hours and precipitating polysaccharides with ethanol, obtaining a total recovery of 5.4% on dry biomass [80]. The extract was then applied at 3 g L^−1^ on tomato and pepper by foliar spraying. The treatments increased shoot dry weight by 140% in both species, while the positive effects on roots weight were much more pronounced in tomato (+230%) than in pepper (+67%) [80]. According to recent findings, lower concentrations (0.25, 0.5, and 1 g L^−1^) of a crude polysaccharide extract from *A. platensis* administered on tomato were also effective in increasing vegetative growth of plants [81]. In this latter study, the extraction was performed at 90 °C with a shorter extraction time (2 h), resulting in a lower recovery of polysaccharides (2.6% on dry biomass). The highest increase in root dry weight (+12% over the controls) and nodes number (+75%) was obtained at 1 g L^−1^, while the highest increase in shoot dry weight (+23%) and shoot length (+13%) was recorded at the lowest concentration (0.25 g L^−1^) [81]. Crude polysaccharide extracts may contain other bioactive metabolites that may contribute to the observed effects. For instance, crude polysaccharides extracted from *Phormidium* *tenue*, composed of 58% carbohydrates and 15% proteins, have been reported to elicit growth and superoxide dismutase activity in seedlings of the shrub *Caragana korshinskii* present in crusted desert areas [63]. Moreover, it has been shown that crude polysaccharides extracted from *A. platensis* contain phenolic compounds (≈ 45 mg gallic acid equivalent g^−1^ of biomass), which display antioxidant activities on plants [150]. According to the authors, the phenolic content significantly increased by increasing extraction temperature from 50 to 90 °C [150].

Taken together, these findings suggest that cyanobacterial polysaccharides may be an effective source of plant biostimulants for crop improvement and protection against abiotic stresses [80]. However, due to the few studies in this field, a direct relationship between the molecular structure of cyanobacterial polysaccharides and their biostimulant activity has yet to be established [149]. Some studies suggest that uronic acids and sulfated groups may be the main polysaccharide constituents at the origin of biostimulation [81,149,151]. As regards the effects on plants, several metabolic pathways, such as photosynthesis and nitrate assimilation, appear to be affected by treatments with cyanobacterial polysaccharides. A significant enhancement in chlorophyll *a* (+90%) and *b* (+102%) content and a concomitant increase in nitrate reductase and NAD-glutamate dehydrogenase activities have been observed in tomato leaves following *A. platensis* crude polysaccharides application [81]. A GC–MS metabolomic analysis also showed an enhancement in phytosterols [81], molecules involved in membrane stability and linked to plant adaptation to temperature variations [152]. The increase in plant sterols could, in turn, lead to the production of brassinosteroids [81], a group of oxidized steroids with hormonal activities responsible for increasing the efficiency of photosynthetic carbon fixation and preventing loss of photosynthetic pigments during stresses [153].

### 3.3. Quality Traits 

The effect of a plant biostimulant cannot be assessed solely on the basis of plant growth as some extracts have demonstrated to trigger biochemical processes that lead to accumulation of important metabolites with consequent improvement of qualitative traits of the final marketable products [59]. Foliar applications of different biostimulants, such as protein hydrolysate, seaweed and plant extracts, were found to improve commercial features (e.g., soluble solids, external color, firmness, fruit size) and nutritional qualities (e.g., phenolic compounds, organic acids, titratable acidity, carotenoids, and anthocyanins) of fruits [32,154,155]. Furthermore, foliar application of biostimulants in some cases reduced the level of undesirable components such as nitrates in greenhouse-grown vegetables [156].

Although cyanobacteria have been shown to be effective in promoting yields in various vegetable and fruit species [45,50,74,83,85,86,90], very few studies have currently examined the effect of cyanobacterial applications on commercial and qualitative traits of the final product. The application of an *A. platensis* extract alone or together with the inoculation of the nitrogen-fixing bacterium *Pseudomonas stutzeri* in the presence of different doses of nitrogen fertilizer, enhanced the growth and productivity of onion under field conditions in two seasons [90]. The best results on bulb yield (marketable yield, total yield, average weight) were obtained with the mixture treatment under the recommended dose of nitrogen. The application of both the *A. platensis* extract alone and the mixture under two different fertilization levels (100% and 75% of the recommended dose) significantly improved bulb quality and conservation, as the treated plants had greater bulb diameter and total soluble solids content and lower cumulative weight loss during storage compared to the respective controls [90]. Opposite results were found in strawberry treated with weekly foliar applications of an *A. platensis* hydrolysate starting from the pre-flowering stage, resulting in lower fruit firmness (–18%) compared to the control and to other biostimulants; moreover, total phenolic and anthocyanin content in fruits was not significantly affected by the *A. platensis* treatment [107]. Salvi et al. [157] found that vines grown in pots and treated with an extract of *A. platensis* F&M-C256 presented, besides a higher berry weight (+11%), total amounts of anthocyanins and polyphenols similar to those of the control, suggesting that the *A. platensis* extract could stimulate the synthesis of these compounds which are mainly located in the berry skin [158]. Furthermore, the treatment influenced sugar loading (+17% mg sugar berry^−1^) over untreated controls [157].

Thanks to their beneficial compounds, such as polysaccharides, polyphenols, antioxidants, and antimicrobials [104,159], cyanobacteria and their extracts can also be useful in post-harvest treatments and in the formulation of edible coatings. For this purpose, *A. platensis* appears to be one of the best candidates since it is authorized almost worldwide as food [102]. The investigation of the physico-chemical qualities of mango fruits coated with guar gum enriched with an aloe vera extract or ethanolic or aqueous extracts of *A. platensis* outlined a strong effect of the ethanolic extract in maintaining a greater fruit firmness during storage compared to control and other coated fruits [160]. The results also indicated that total phenolic content and radical scavenging activity were much higher in fruits coated with guar gum and the ethanolic extract, while ascorbic acid content reached the highest values with guar gum and the aqueous extract [160].

Foliar applications of cyanobacterial extracts may have an effect in modulating nutritional and functional properties of the final marketable product. Oil and NPK content of fenugreek seeds was markedly increased in plants treated with an *A. platensis* extract applied at two different concentrations [85]. The highest increase (+90%) in oil content compared to untreated plants was recorded in seeds treated with the highest extract concentration (5 g L^−1^) [85]. A comparable increase in oil (+77%) was also found in cardoon seeds subjected to foliar applications of *A. platensis* extracts at similar or lower concentrations (1, 2, and 3 g L^−1^) [87]. Foliar spraying with a water suspension of *Nostoc entophytum* MACC-612 at 0.3 g L^−1^ significantly increased the seed yield of winter rapeseed without affecting the oil content [60]. Total carotenoid, tocopherol, phenolic, and protein contents in whole grains of wheat irrigated with 10 and 20% (v/v) seawater were significantly increased in response to application of water extracts of *Spirulina maxima* and *Chlorella ellipsoidea* [67]. However, under certain conditions, *S. maxima* performed better than *C. ellipsoidea* in increasing phenols and tocopherol compared to controls irrigated with diluted seawater only. Radical scavenging activity of grains was in accordance with the increased levels of antioxidant compounds [67].

Very few studies have currently explored the application of cyanobacteria in improving qualitative traits of medicinal plants. Peppermint is a widespread perennial aromatic herb, known for containing many essential oils (menton, menthol, pulegone, and carvone) with several applications in food, cosmetics, and pharmaceutics [161]. The application of extracts obtained from *A. vaginicola* and *Cylindrospermum michailovskoense* as a soil spray has been shown to enhance the number and size of peppermint leaf (on average by 128% and 112%) and to increase the essential oil content in the leaves up to 60% compared to controls sprayed with water [61].

Inoculation with cyanobacteria has been also proposed as a strategy for biofortification of staple crops [71,89,162]. Wheat seeds inoculated with a consortium of *Anabaena* sp. and *Calothrix* sp. brought about a 13% increase in seed protein content and a concomitant enhancement in micronutrient concentration (Zn and Cu) when compared to the application of chemical fertilizer alone [71]. Evidence suggests that nitrogen nutritional status of plants can have a positive impact on root–shoot translocation of nutrients and on re-translocation of micronutrients from vegetative tissues into seeds [71,163]. Therefore, the ability of cyanobacteria to increase nutrient use efficiency can ultimately improve the nutritional quality of grains.

## 4. Cyanobacteria in the Biostimulant Market: Current Status and Main Criticalities

Although there seems to be opportunities to largely exploit cyanobacteria as plant biostimulants, few well-characterized products are currently on the market, most of which are based on *Arthrospira* spp., with Spain being the leading EU country in the production of cyanobacterial biostimulants (Table 2). In other registered products the species used are not indicated (Table 2). In the large and heterogeneous market of biostimulants, cyanobacteria-based products still represent a very small niche, especially when compared to the multitude of seaweed-based products that constitute more than 33% of the total market worldwide [18].

The exploitation of cyanobacteria for plant biostimulants could be hampered by the fact that these microorganisms are currently not included in the EU regulation 2019/1009 that will come into force in 2022 [11] and by the fact that several species of cyanobacteria produce toxins [164] whose presence must be carefully assessed before using cyanobacteria on crops. Moreover, compared to seaweeds, which are collected from marine waters offering biomass at very competitive costs (ranging from an average of € 0.6 kg^−1^ dry weight in Asia and South America to € 3–15 kg^−1^ dry weight in Europe) [165,166], cyanobacteria, and also microalgae, are usually cultivated in controlled and confined systems (photobioreactors and open ponds), thus constituting a more expensive source of biomass for the production of plant biostimulants. In fact, these cultivation systems require significant amounts of energy and matter inputs, mainly in the form of electrical power, fertilizers, water, and materials for production facilities construction [167,168]. Estimated production costs of cyanobacterial and microalgal biomass produced in commercial facilities range from about € 5 to over € 860 kg^−1^ [169] based on the country of origin and the cultivation method adopted (natural or artificial light, photobioreactors, or open ponds). The cost of production in commercial closed reactors averages € 50 kg^−1^, much depending on the productivity and requirements of the species cultivated [170]. However, production costs ranging from € 3.2 to € 12.4 kg^−1^ can be achieved for microalgae produced at large scale in closed systems (GWP^®^-II) under natural light and favorable climatic conditions [170]. On the other hand, the use of controlled cultivation conditions along with the plasticity of cyanobacterial and microalgal metabolism provides a wide range of possibilities to improve quality and standardize biostimulants. Different light qualities and high light intensities have been shown to promote polysaccharide production in cyanobacteria, for example in *Nostoc flagelliforme* red light maximized the effects [171]. In natural light conditions, it is possible to significantly influence abscisic acid, auxin, and salicylic acid contents in cyanobacterial biomass by manipulating culture inoculation density and consequently light availability to the single cell [172].

According to our market survey, the treatment cost can vary, depending on application doses and number of applications, between € 20 and € 375 per hectare (Table 2), the upper part of the range possibly being not affordable for some farmers. To make biostimulants from cyanobacteria more competitive it will be necessary to reduce biomass production costs, for instance by integrating the cultivation with wastewaters treatment (removal of N and P), by using CO_2_ from waste streams or by using thermotolerant strains that require no cooling [170,173,174]. Besides, to improve the environmental sustainability of the whole process, the production of cyanobacteria can be integrated with the use of renewable energy sources (e.g., photovoltaic and geothermal) [167,175]. Ideally, the production of biostimulants can be coupled with the production of other desirable products from cyanobacteria adopting a biorefinery approach. However, this requires that the molecules that most contribute to the biostimulant action are identified, in order to evaluate the possible re-uses of the remaining fractions. Among the available possibilities, the residual pellet after extraction could be used as a biofertilizer [82]. If aqueous cellular extracts are the main biostimulants components, the remaining lipid fraction could be used for the production of biofuels [176] or to obtain polyunsaturated fatty acids (PUFAs) which have several cosmetics, medical, and nutraceutical applications [164], or polyhydroxyalkanoates (PHA) which could be used for the production of bioplastics [177]. Moreover, when polysaccharides are used to obtain the biostimulant, the residual proteins can find application as food or feed [164].

## 5. Concluding Remarks and Challenges Ahead

Cyanobacteria are receiving increasing interest from the scientific community and the agrochemical industry as a new renewable source of plant biostimulants capable of sustainably improving yields and quality of agricultural and ornamental crops. The adoption of cyanobacterial biostimulants in agriculture could allow for the production of more from the same area of land while reducing dependency on synthetic fertilizers and supporting the shift towards a sustainable intensification of agriculture.

Plant inoculation and seed coating with living cyanobacteria or application of cyanobacterial extracts or extracellular products have shown several beneficial effects including improved seed germination, seedling growth, flowering, photosynthetic activity, nutrient use efficiency, and tolerance to abiotic stresses, thereby optimizing plant productivity in stressed and unstressed conditions. These effects are attributed to a diversity of biologically active molecules produced by cyanobacteria such as phytohormones, amino acids, proteins, antioxidants, carbohydrates, and polysaccharides. The numerous in vivo trials conducted so far on different plant species evidence that cyanobacteria have the characteristics to meet the definition of a “plant biostimulant” by the EU regulation. However, it is difficult to directly compare the effects of the application of different cyanobacterial strains or even the same strain on different plant species as various extraction methods, doses, times, and modes of application are used and different parameters are evaluated. Therefore, the drafting of standard protocols for the validation of new biostimulants is highly recommended to facilitate the entry into the market of new products.

Even if the development and marketing of novel biostimulants currently do not require a clear demonstration of the mode of action, to maximize the beneficial effects of cyanobacterial applications a better understanding of how different strains specifically influence plant physiology is needed. Experimental evidence suggests that there is a relationship between bioactive compounds produced by cyanobacteria and the effects on plant physiology. However, there is a lack of studies that clearly identify the role and relevance of the different molecules involved in the biostimulation process. Although biostimulant benefits are usually considered as “the consequence of the emergent properties of a complex of constituents”, we cannot exclude the possibility of antagonistic interactions, which could result in reduced effects on plant growth compared to purified compounds. The search for biostimulant mechanisms is further complicated by the fact that many of the feedstocks currently in use (e.g., seaweeds and agro-industrial by-products) may have a variable composition intrinsically related to their production processes, which may hinder the reproducibility of the effects on treated species. The use of cyanobacteria can provide a solution to these problems that undermine the biostimulant market as it could allow to obtain raw materials under highly regulated conditions, ensuring standardization and safety of the final product.

The ultimate price of the product will be the key factor determining the future use of cyanobacterial formulations in agriculture. Compared to the use as biofertilizers, the application at low doses configures a greater remunerability of the use of cyanobacteria as biostimulants which compensates for the high production costs. However, several biological, agronomical, economic, and technological issues must be solved before such products become widely diffused on the market:

- optimization of cultivation conditions for the production of the target molecules and design of specific extraction procedures for the preservation of high levels of bioactive substances in the final product;
- reduction of cyanobacterial biomass production costs through the use of innovative and efficient cultivation systems and biorefinery approaches;
- reduction of application costs through in-depth studies on application methods (e.g., minimum active doses, time of application) according to the plant species.

Therefore, the adoption of a multidisciplinary approach, including discovery of new bioactive strains, optimization of cultivation conditions, biochemical characterization of the biostimulant, understanding of the mechanisms of action, and extensive agronomic trials will be fundamental to develop cyanobacterial biostimulants adapted to specific crops and environments.

## Figures and Tables

**Table 1:** Literature review on the application of cyanobacteria on various plant species and main effects produced.

Genus/Species	Positive Effects (Increase of the Reported Feature)	Plant
*Scytonema hofmanii*	tolerance to salt stress [58]	rice [58]
*Aphanothece* sp.	plant growth [59] macro- and micronutrient content [59]	tomato [59]
*Tetracystis* sp.	plant growth [60] pigment content [60]	rapeseed [60]
*Cylindrospermum* sp.	plant growth [61,62] pigment content [62] essential oil content [61]	peppermint [61] lupin [62]
*Phormidium* sp.	plant growth [43,63] antioxidant activity [43,63]	rice [43] *Caragana korshinskii* [63]
*Microcystis aeruginosa*	plant growth [64] pigment content [64] macro- and micronutrient content [64] photosynthesis rate [64] tolerance to thermal stress [65]	willow [64] *Sida hermaphrodita* [65]
*Oscillatoria* sp.	plant growth [43] pigment content [66] antioxidant activity [43] carbohydrate and protein content [66]	rice [43] sunflower [66]
*Arthrospira maxima*	antioxidant activity [67]	wheat [67]
*Arthrospira fusiformis*	bulb diameter [68]	garlic [68]
*Calothrix* sp.	plant growth [43,61,69,70] macro- and micronutrient content [71] germination [72] seed yield [73] essential oil content [61] phytohormones content [69] antioxidant activity [43,70]	rice [43,69] coriander [70] peppermint [61] wheat [71,73] cotton [72]
*Nostoc* sp.	plant growth [74,75,76] seed yield [60,73,74] pigment content [74,75,76] tolerance to cold and drought stress [77] leaf relative water content [74]	rapeseed [60] wheat [73,74] cantaloupe [75] lettuce [76] bean [77]
*Anabaena* sp.	plant growth [43,54,61,62,64,75,76,78] germination [70,72] macro- and micronutrient content [64,71] pigment content [62,64,75,76,78] photosynthesis rate [64] essential oil content [61] phytohormones content [78] antioxidant activity [43] tolerance to thermal stress [65] reduction of heavy metal content [54]	rice [43] spinach [54] peppermint [61] lupin [62] willow [64] *Sida hermaphrodita* [65] wheat [71] cotton [72] cumin [70] cantaloupe [75] lettuce [76] chrysanthemum [78]
*Arthrospira platensis*	plant growth [50,79,80,81,82,83,84,85,86,87,88] macro- and micronutrient content [53,79,83,84,85] pigment content [88] essential oil content [85,87] carbohydrate and protein content [89] vitamin A content [89] phytohormones content [88] earliness of flowering [84] reduction of flower abortion [82] bulb yield, quality, and storage [90] tolerance to salt stress [91] tolerance to cadmium toxicity [92] spermine content [50]	lettuce [50,79] mung bean [53] tomato [53,80,81,82] pepper [80] radish [83] fenugreek [85] red beet [86] cardoon [87] cotton [88] amaranth [53,89] onion [90] petunia [84,91] maize [92]

**Table 2:** Cyanobacterial biostimulants currently on the market. Products claimed as microalgae-based have been included in the survey when the species used were not indicated in the product information as the term microalgae often includes cyanobacteria. To distinguish biostimulants from fertilizers, only products whose benefits are attributed to the content of phytohormones, free amino acids, and other bioactive molecules were considered. The average treatment costs were calculated on the basis of the doses recommended on the label for horticultural and fruit crops and without considering the costs necessary for equipment and labor required to perform the treatment.

Brand	Company	Species	Average Price	Mode of Application	Average Treatment Cost
Spiragro Spiragrow	Neoalgae Micro Seaweeds Products (Spain)	*Arthrospira* *platensis*	10 € L^−1^	foliar and radical	20–50 € ha^−1^
Floralgal Algafert	Biorizon Biotech (Spain)	*Arthrospira* sp.	/	foliar and radical	/
Shwe Awzar Spirulina	June Industry Limited (Myanmar)	*Arthrospira* sp.	/	radical-soil conditioner	/
Microp	Soiltech (USA)	unspecified cyanobacteria	0.27 € g^−1^	radical-soil conditioner	23–91 € ha^−1^
Agrialgae^®^	AlgaEnergy (Spain)	unspecified microalgae	25 € L^−1^	foliar and radical	125–375 € ha^−1^
Ferticell	Agroplasma (Spain)	unspecified microalgae and bacteria	15 € L^−1^	foliar and radical	/
Phycoterra	Heliae development LLC (USA)	unspecified microalgae	/	radical	/

## Data Availability

Not applicable.
